# The Influence of Input Frequency and L2 Proficiency on the Representation of Collocations for Chinese EFL Learners

**DOI:** 10.3390/bs15010046

**Published:** 2025-01-04

**Authors:** Mengchu Yu, Saisai Xu, Lianrui Yang, Shifa Chen

**Affiliations:** School of Foreign Languages, Ocean University of China, Qingdao 266005, China; xusaisai@stu.ouc.edu.cn (S.X.); lryang@ouc.edu.cn (L.Y.); chenshifa99@ouc.edu.cn (S.C.)

**Keywords:** input frequency, L2 proficiency, collocational representation, continuum pattern, Chinese EFL learners

## Abstract

Collocations typically refer to habitual word combinations, which not only occur in texts but also constitute an essential component of the mental lexicon. This study focuses on the mental lexicon of Chinese learners of English as a foreign language (EFL), investigating the representation of collocations and the influence of input frequency and L2 proficiency by employing a phrasal decision task. The findings reveal the following: (1) Collocations elicited faster response times and higher accuracy rates than non-collocations. (2) Higher input frequency improved the accuracy of judgments. High-proficiency Chinese EFL learners exhibit better accuracy and faster response times in collocation judgment tests. Additionally, input frequency and L2 proficiency interactively affected both response time and accuracy rate. These results indicate that L2 learners have a processing advantage for collocations, which function as independent entries in the mental lexicon. Both input frequency and L2 proficiency are crucial factors in collocational representation, with increased input frequency and proficiency shifting the representation from analytic retrieval toward holistic recognition in a continuum pattern.

## 1. Introduction

Collocation is indicative of the horizontal relationship between words, integrating the lexical, grammatical, semantic, and pragmatic knowledge of languages, playing a crucial role in language use and cross-linguistic communication (Nation, 2001). It is widespread in texts and reflects the construction and development of lexical networks of language users’ mental lexicons (Ellis et al., 2009). In linguistic systems, collocations function as essential units of language processing, with their distributional information identifiable throughout comprehension and production (Siyanova-Chanturia, 2015). Most research on collocations has primarily focused on native speakers, with limited attention to second language (L2) learners. Recent studies have shifted the focus to investigating the L2 mental lexicon, particularly how L2 collocations are represented under the influence of various linguistic and non-linguistic factors (Cao, 2016; Gyllstad & Wolter, 2016; Öksüz et al., 2024). However, there is ongoing debate regarding whether collocations are represented holistically or analytically in the mental lexicons of Chinese EFL learners. Moreover, the impact of input frequency and L2 proficiency remains under-explored. On the one hand, the frequency of collocation extracted from the native speaker corpus lacks representativeness in reflecting the input of L2 collocations for Chinese EFL learners. On the other hand, the current studies are heavily skewed toward adult learners with higher L2 proficiency (especially undergraduate and graduate students), while adolescent learners with lower proficiency have received little attention. Therefore, utilizing a corpus-based psycholinguistic paradigm and extracting collocation frequency from a self-constructed corpus of English textbooks for Chinese EFL learners, this study aims to examine the impact of input frequency and L2 proficiency on the representation of collocations, particularly targeting both lower-proficiency adolescent and higher-proficiency adult Chinese EFL learners. Collocations with consistent meaning and form in both English and Chinese are focused. For instance, the English phrase “similar concept” corresponds to the Chinese “相似的概念”. Both of them constitute adjective–noun word pairs in two languages, with “相似的” and “概念” serving as the translation equivalents of “similar” and “concept”, respectively.

## 2. Literature Review

Collocations have long been a central topic in linguistic research, garnering significant scholarly attention and resulting in a substantial body of work. Although the term “collocation” was first introduced in the 1930s (Palmer, 1933), it was J. R. Firth who clearly defined and elaborated it in 1957. Firth distinguished collocation from grammar, defining it as the habitual juxtaposition of lexical items, which refers to the co-occurrence of words. His famous assertion, “You shall know a word by the company it keeps” (Firth, 1957, p. 179), underscores the importance of collocation. Afterward, scholars have approached collocations from the lexical, structural, and semantic perspectives, leading to diverse definitions and classification systems. Two dominant approaches to defining collocations have emerged in the state-of-the-art literature: the phraseological approach and the frequency-based approach (Gablasova et al., 2017; Howarth, 1998; McEnery & Hardie, 2012; Nesselhauf, 2005; Öksüz et al., 2024). The former emphasizes semantic associations between words which contribute to compositional meaning (Howarth, 1998; Nesselhauf, 2005), while the latter draws on quantitative evidence of word co-occurrence in corpora within a limited range (Gablasova et al., 2017; McEnery & Hardie, 2012; Öksüz et al., 2024).

In the realm of theoretical inquiry, a central debate revolves around how language users cognitively process collocations. Generative linguistics and functional linguistics provide contrasting explanations regarding whether multi-word sequences are processed in the same way as single words. The generative approach (Chomsky, 1995; Pinker, 1999) advocates a dual-system model, suggesting that lexical knowledge and grammar are represented separately in the brain. The storage and retrieval of lexical knowledge rely on declarative memory, while multi-word sequences (including collocations) that involve the use of rule-governed aspects of grammar are supported by procedural memory (Ullman, 2001; Ullman, 2004). In contrast, the usage-based approach (Bybee, 2007; Langacker, 1987) denies the existence of abstract rules that are independent of language use. It is believed that language acquisition is not the learning of words and rules, but rather a process of abstracting frequency-oriented language patterns based on language experience. The usage-based approach supports a single-system model, arguing that both single words and larger linguistic units are processed through the same cognitive mechanism.

The usage-based approach has received substantial empirical support, particularly from studies examining how native speakers process multi-word sequences. The findings indicate that multi-word sequences are holistically represented in the mental lexicon of native speakers (Jiang et al., 2020; Tremblay et al., 2011). As the co-occurrence frequency of words in multi-word sequences increases, the entrenchment of the word strings as a whole in psychological representation strengthens. Native speakers exhibit sensitivity to multi-word co-occurrence frequency in both language comprehension (Hernández et al., 2016; Siyanova-Chanturia et al., 2011) and production (Arnon & Priva, 2013, 2014). Thus, collocation can be regarded as the fundamental unit in language processing and accelerator of language comprehension and production for native speakers.

Research on L2 learners indicates that fixed language forms in multi-word sequences, such as lexical bundles (e.g., in the middle of) and binomials (e.g., knife and fork), are not only ubiquitous in texts but also have psychological reality. In contrast, collocations represent a relatively flexible form of lexical combination, wherein the constituent words can form new collocations with other words. However, while collocational components exhibit a degree of flexibility, they also possess certain constraints, which pose greater challenges for language acquisition. Therefore, how can collocations be represented in the mental lexicon of L2 learners has become the key issue. Along this line, Cao (2016) argued that the representation of collocations was closely linked to learners’ L2 proficiency, with low-proficiency learners totally relying on analytic representation and high-proficiency learners employing both holistic and analytic representation. In other words, only advanced L2 learners can fully acquire collocation knowledge, while words tend to remain isolated in the mental lexicon of low-proficiency L2 learners, preventing lexical connections. These L2 proficiency-related differences in the representation of collocations were significant. However, contrary to this view, evidence from the studies by Wolter and Yamashita (2017), and Fang and Zhang (2019) showed that both the component word and the entire collocation could be activated simultaneously for high- and low-proficiency L2 learners, indicating the coexistence of holistic and analytic representation, with high-proficiency learners showing a greater reliance on the holistic representation. Thus, learners at different proficiency levels can engage in top-down processing, with differences mainly reflecting varying degrees of automaticity. In addition to L2 proficiency, collocation frequency plays a significant role in the representation of collocations. Collocation frequency moderates the processing channels, with L2 learners favoring holistic representation for high-frequency collocations and analytic representation for low-frequency collocations (Lou, 2022; Zhang & Fang, 2020). Furthermore, the sensitivity to collocation frequency varies among learners of different L2 proficiency levels. Studies by Gyllstad and Wolter (2016), Wolter and Yamashita (2017), and Zhang and Fang (2020) suggested that L2 learners’ sensitivity to collocation frequency increased with proficiency, potentially reaching native-speaker levels. Conversely, Sonbul (2015) found that L2 learners exhibit limited sensitivity and struggle to perceive collocation frequency in L2 input, with the frequency effect remaining stable regardless of proficiency levels.

Given the mixed findings, the representation of collocations in L2 learners’ mental lexicon is unclear, particularly regarding how input frequency and L2 proficiency shape the representation of collocations. Additionally, the majority of the existing studies have focused on adult L2 learners with high proficiency, largely overlooking adolescent L2 learners with low proficiency. Furthermore, prior studies have typically extracted language materials and frequency indicators from native-speaker corpora (e.g., COCA, BNC, etc.), which may not align with the input that L2 learners are exposed to. In response, this study draws on a self-constructed corpus of English textbooks for Chinese learners (CETCL), focusing on both adolescent and adult Chinese EFL learners, to investigate the effects of input frequency and L2 proficiency on the representation of collocations. Specifically, the research questions are:

(1) How do Chinese EFL learners represent collocations in the mental lexicon?

(2) To what extent does the frequency of occurrence of particular collocations in the input and learners’ L2 proficiency influence the representation of collocations by Chinese EFL learners?

## 3. Method

This study adopted a two-factor mixed design using a phrasal decision task, with collocation frequency (low-frequency/high-frequency) as the within-subject variable and English proficiency (low/high) as the between-subject variable. Response time and accuracy rate were treated as the dependent variables.

### 3.1. Participants

Participants were recruited from a high school and a university in mainland China. To ensure the validity of the study and the comparability between the two groups, the sample comprised 40 third-year undergraduate English majors and 40 12th-grade high school students (the last year of high school in China). All the participants are Mandarin native speakers learning English as a second language, with 25 males and 55 females (mean age = 19.76). They participated in the experiment after completing their English courses in the semester. Prior to the main experiment, all the participants signed an informed consent form and filled out a personal information questionnaire (see Supplementary Table S1). This procedure ensured voluntary participation, assured the confidentiality of their personal information and responses, and collected demographic information, including age, gender, grade, starting age of learning English, length of formal English education, duration of residence in English-speaking countries, etc.

Subsequently, the Oxford Quick Placement Test (OQPT) was employed to determine group assignment. Based on OQPT placement criteria, 32 high school participants were assigned to the low-proficiency non-native speakers (hereafter, LNNS) group, and 34 university participants were assigned to the high-proficiency non-native speaker (hereafter, HNNS) group. To verify the comparability between the groups in terms of learning-related variables, independent samples *t*-tests were conducted. The results showed no significant differences in the participants’ starting age of learning English (*t* (64) = −1.16, *p* = 0.25). However, significant differences were found in the length of English learning experience (*t* (64) = −7.99, *p* < 0.001) and OQPT scores (*t* (64) = −9.17, *p* < 0.001), demonstrating that HNNS had a longer history of English study and a higher proficiency than LNNS. All the participants were right-handed with natural or corrected-to-natural vision, ensuring consistency in motor and visual conditions that could influence experimental performance. Only one participant reported prior experience traveling in an English-speaking country, and this exposure was limited to less than one month, minimizing the impact of immersive language experience on the study. The participants were compensated upon the completion of the experiment as a gesture of appreciation for their time and effort. Table 1 summarizes the participants’ background information.

### 3.2. Item Development

This study adopted a frequency-based approach to define collocations, focusing on the co-occurrence of two words that are semantically transparent, structurally compliant, and non-random. Semantic transparency implies that the meaning of a collocation is derived directly from the meanings of its component words (e.g., blue sky), in contrast to the opaque collocation (e.g., blue tooth). Structural compliance refers to the collocation conforming to grammatical rules. Non-random co-occurrence means that the score of mutual information (MI) between the component words in a corpus is not less than 3 (Yi, 2018; Yi et al., 2022).

The experimental materials were derived from a self-constructed corpus of English textbooks for Chinese learners (CETCL), which represents L2 input and contains a total of 1,422,612 word tokens. Prior to the construction of the CETCL, a questionnaire was administered to collect data on the sources of L2 input and the editions of English textbooks for Chinese EFL learners. Ultimately, a total of 40 textbooks were selected, as textbook-based L2 input was reported by over 80% of the respondents. The CETCL encompassed all the content encountered by Chinese EFL learners across textbooks, including passages, exercises, and vocabulary lists, from primary school to college. The content was first extracted and converted into “.txt” format using the ABBYY FineReader software (version 12), followed by annotation with the TreeTagger software (version 3) and manual verification. The CETCL was designed to contain four sub-corpora, namely the corpus of textbooks for Chinese primary students, the corpus of textbooks for Chinese middle school students, the corpus of textbooks for Chinese high school students, and the corpus of textbooks for Chinese college students in English majors. To ensure consistency in parts of speech, adjective–noun collocations were extracted, as these combinations tend to exhibit less variability in determiners compared to verb–(object)–noun combinations (e.g., make a mistake vs. make progress) (Wolter & Gyllstad, 2013). Then, to establish frequency bands, non-lemmatized frequency counts were utilized at both the lexical and collocational levels, with the threshold set at over 10 occurrences per million for high-frequency collocations and under 5 occurrences per million for low-frequency collocations. Based on the results obtained from this step, 200 adjective–noun collocations were randomly selected as the candidate materials.

Next, two questionnaires were adopted to minimize interference from familiarity and semantic congruency. The first questionnaire assessed the participants’ familiarity with high- and low-frequency collocations, involving 8 students who did not participate in the main experiment (4 12th-grade high school students and 4 third-year undergraduate English majors). The second questionnaire evaluated the semantic congruency of the collocations and was completed by 4 PhD students majoring in Applied Linguistics. These processes further refined the candidate materials to include only those with familiar and congruent collocations (i.e., English collocations that have translation equivalents in Chinese, e.g., similar concept), with the mean scores greater than 4 on a five-point Likert Scale.

Following this, 60 target adjective–noun collocations for each group of participants were selected, comprising 30 high-frequency and 30 low-frequency collocations. These collections were structurally consistent and semantically equivalent between Chinese and English. The target collocations for the LNNS group were derived from the corpus of textbooks for Chinese high school students (including content in English textbooks from primary school to high school), while those for the HNNS group were derived from the corpus of textbooks for Chinese college students in English majors (covering content in English textbooks from primary school to university). Both types of collocations consisted of 8 to 14 characters, with no significant differences in length (i.e., the total number of letters for collocations; LNNS: *t* (58) = −1.30, *p* = 0.20; HNNS: *t* (58) = −1.16, *p* = 0.38). This was true for noun frequency (frequency per million; LNNS: *t* (58) = −1.56, *p* = 0.11; HNNS: *t* (58) = −1.34, *p* = 0.18) and adjective frequency (frequency per million; LNNS: *t* (58) = 1.63, *p* = 0.11; HNNS: *t* (58) = −0.06, *p* = 0.96), but showed a significant difference between collocation frequency (frequency per million; LNNS: *t* (58) = −6.99, *p* < 0.001; HNNS: *t* (58) = −7.86, *p* < 0.001).

Ultimately, 60 non-collocations were selected as baseline items for the phrasal decision task. These items consisted of word combinations containing either grammatical errors (e.g., deeply hole) or semantic anomalies (e.g., broad price), with constituent words drawn from the top 5000 words in the COCA, ensuring high familiarity for the participants (Milton, 2009). To eliminate potential interference from word repetition and inconsistency in the length of collocations and non-collocations, no constituent words from the target collocations appeared in the non-collocation baseline items, and the string length of the baseline items adhered to the same range as the collocations (*M* = 11.05, *SD* = 1.32). The classification of non-collocations was verified by individually checking each baseline item in the COCA to confirm that the non-collocations failed to occur in the corpus or their MI scores were smaller than 1, suggesting that the expressions are peculiar rather than significant co-occurrences in English (Wolter & Yamashita, 2017). A complete description of the items is provided in Table 2, and the research items adopted in the phrasal decision task are listed in Supplementary Table S2.

### 3.3. Procedures

A phrasal decision task was employed, requiring participants to make a judgment about whether the given item was a possible sequence in English, consistent with the method employed by Hernández et al. (2016). The task was administered using the E-prime software (version 3.0). All the items were presented on a computer screen in an individually determined, randomized order. The participants began the experiment by reading the instructions displayed on the computer screen and pressing the space bar to start. Ten practice trials were provided to familiarize the participants with the procedure, and only after confirming their understanding did they proceed to the main trials.

**Figure 1:**
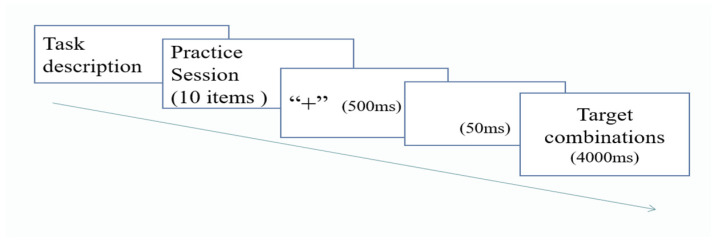
Procedure of the phrasal decision task.

The procedure for each block of trials was as follows: a red fixation point “+” appeared at the center of the computer screen for 500 milliseconds (hereafter, ms) to alert the participants, followed by a blank screen for 50 ms. When the target item appeared on the screen, the participants were required to make a judgment within 4000 ms before the next trial began. The participants needed to quickly assess whether the test item represented a correct English collocation and respond using the keyboard. If they considered the target item a valid English collocation, they pressed the “J” key with the right hand; otherwise, they pressed the “F” key with the left hand. The item disappeared automatically if no response was made within 4000 ms. To prevent the fatigue effect, the participants took a self-defined time break after every 60 items. The participants completed the task independently in a quiet computer lab, taking approximately 8 min to finish it. The task procedure is visualized in Figure 1.

## 4. Analysis and Results

Data from 66 participants, comprising a total of 7920 items, were automatically recorded by the E-prime software (version 3.0) and then stored in Excel files. The dataset includes both accuracy rate (hereafter, ACC) and reaction time (hereafter, RT) data. Before fitting the model, data trimming was performed following three steps: (1) removing the participants’ data with ACCs below 60%; (2) eliminating RT data linked to incorrect judgments; (3) excluding outliers of RT data below 450 ms, above 4000 ms, and those exceed the mean by 2.5 standard deviations. In total, 333 data items were removed, representing 3.28% of the trials in the HNNS group and 5.18% in the LNNS group. The descriptive statistics obtained for the trimmed RT and ACC data in the phrasal decision task are listed in Table 3.

The trimmed data were then imported into the R statistical platform (version 4.2.2), where the RTs were log-transformed, and all the numeric variables were centered and standardized prior to the analysis. A top-down approach, specifically backward stepwise regression, was utilized for model establishment. Linear mixed-effects models were established using the lmer() function for the RT data, while generalized linear models were established for the ACC data using the glm() function (Kuznetsova et al., 2017). And post hoc tests were conducted using the emmeans() function (Naimi et al., 2014).

Based on the participants’ processing of collocations versus non-collocations, mixed-effects model I for RTs and generalized linear model I for ACCs were established, with the results presented in Table 4 and Table 5, respectively. In these models, both the participants and items were treated as random variables, while type served as a fixed effect. For RTs, the result of mixed-effects model I indicated a main effect of type (*β* = 0.48, *t* = 39.83, *p* < 0.001), revealing that the participants processed collocations significantly faster than non-collocations. Similarly, the result of generalized linear model I for ACCs demonstrated a main effect of type (*β* = −1.36, *z* = −17.22, *p* < 0.001), indicating that the participants had significantly higher ACCs in judging collocations compared to non-collocations. These findings highlight the processing advantage for L2 collocations over non-collocations.

Based on the participants’ judgment responses to collocations, mixed-effects model II for RTs and generalized linear model II for ACCs were established, with the results presented in Table 6 and Table 7, respectively. In these models, both the participants and items were treated as random variables, while group, collocation frequency, and their interactions served as fixed effects.

For RTs, the result of mixed-effects model II exhibited a significant main effect of group (*β* = 0.13, *t* = 2.66, *p* = 0.01), showing that LNNS had significantly longer RTs when judging collocations compared to HNNS. Additionally, there was a significant interaction effect between the group and collocation frequency (*β* = 0.08, *t* = 10.68, *p* < 0.001). The post hoc analysis revealed that LNNS processed high-frequency collocations significantly faster than low-frequency collocations (*β* = −0.07, *t* = −6.86, *p* < 0.001), while there was no difference in RTs between the high- and low-frequency collocations for HNNS (*β* = 0.01, *t* = 1.17, *p* = 0.24). In both the high-frequency (*β* = −0.13, *t* = −2.66, *p* = 0.01) and low-frequency (*β* = −0.21, *t* = −4.41, *p* < 0.001) conditions, the differences between LNNS and HNNS were statistically significant.

For ACCs, the result of generalized linear model II indicated a significant main effect of the group (*β* = −1.29, *z* = −4.59, *p* < 0.001), with LNNS showing lower ACCs in judging collocations compared to HNNS. The main effect of collocation frequency was also significant (*β* = −1.44, *z* = −4.62, *p* < 0.001), indicating that the participants had significantly lower ACCs in processing low-frequency collocations compared to high-frequency collocations. Furthermore, there was a significant interaction between the group and collocation frequency (*β* = 1.13, *z* = −3.08, *p* = 0.01). The post hoc analysis revealed that LNNS showed no significant differences in ACCs between the high- and low-frequency collocations (*β* = 0.31, *z* = 1.62, *p* = 0.11), whereas HNNS demonstrated significantly higher ACCs for the high-frequency collocations compared to low-frequency collocations (*β* = 1.44, *z* = 4.62, *p* < 0.001). In the high-frequency condition, the difference between the two groups was significant (*β* = 1.29, *z* = 4.59, *p* < 0.001), with HNNS achieving higher ACCs; however, no significant difference was observed between the groups in the low-frequency condition (*β* = 0.16, *z* = 0.70, *p* = 0.48). These results suggested that both L2 proficiency and collocation frequency influence the representation of collocations in the L2 mental lexicon.

## 5. Discussion

### 5.1. The Representation of L2 Collocations

This study found that, compared to non-collocations, Chinese EFL learners exhibited shorter reaction times and higher accuracy rates for collocations, demonstrating a clear processing advantage for collocations over non-collocations. Similar findings have been reported in other studies, despite the use of different research paradigms (e.g., phrasal decision task (Arnon & Snider, 2010), eye-tracking studies (Sonbul, 2015), and ERP studies (Hughes, 2018)) and the focus on L2 learners with varied native language backgrounds (e.g., Swedish learners of English (Wolter & Gyllstad, 2013) and Turkish learners of English (Öksüz et al., 2024)). These results suggest that collocations have distinct entries in learners’ L2 mental lexicon, suggesting that words in collocations are stored holistically. The holistic representation of collocations allows for their simultaneous retrieval from memory during processing, eliminating the need for the separate activation of component words and the synthesis of grammatical knowledge. The finding of this research expanded the understanding of multi-word sequences, such as lexical bundles (Tremblay et al., 2011) and binomials (Sonbul et al., 2023) in previous research, to collocations. It indicated that despite the relatively low degree of fixedness of collocations, which reflects the flexibility of language, collocations exhibit independent accessibility as a whole. The psychological reality of collocations corresponds to the principles of the usage-based theory of language acquisition and processing. The acquisition of collocations, similar to other skills, relies on general cognitive abilities, such as categorization, chunking, analogy, and rich memory storage, rather than from innate language acquisition mechanisms. For Chinese EFL learners, repeated exposure to numerous language exemplars helps reinforce and abstract regular language forms. Linguistic constructions then emerge through natural language use and are cognitively represented in the brain. The basic unit of language is not the word but the construction. Therefore, collocations, in contrast to non-collocations, are reinforced through repeated exposure, making them easier to store, recall and retrieve. Collocations, along with larger linguistic units and even sentences, are all considered constructions due to their conventionalized form and meaning, thereby possessing psychological reality in language users’ mental lexicon (Sonbul et al., 2023).

Additionally, the processing advantage of collocations over non-collocations can be explained by the transitional probability of words in English (McDonald & Shillcock, 2003; Valsecchi et al., 2013), as well as the differences in collocational formation between Chinese and English. The frequent co-occurrence of two words aids language users in predicting collocational components, facilitating the activation between words and reducing the processing time for collocations (Öksüz et al., 2024). In contrast, non-collocations, as mentioned in the material selection section, lack the probability of co-occurrence in the input. Therefore, Chinese EFL learners must undergo a process of forming predictions about collocational components, experiencing mismatches, followed by a stage of retrieval of word items from the mental lexicon, ultimately leading to judgment behavior. The low-transitional probability of words in non-collocations increases the cognitive load, as evidenced by slower processing speeds and lower accuracy rates in this study. Additionally, it is further supported by longer reading times and higher fixation counts in eye-tracking studies (Sonbul, 2015), as well as the N400 components responding to semantic violations for non-collocations in ERP studies (Hughes, 2018). From the perspective of linguistic typology, the frequently examined collocations share the same structural patterns in both Chinese and English (e.g., noun–adjective, verb–noun, noun–noun, and adverb–adjective collocations) (Fang & Zhang, 2019). However, the verb–preposition combinations involved in this study are inconsistent in Chinese. For instance, in Chinese, the verb “arrive” can directly connect with a place without the need for prepositions such as “in” or “at”. The absence of such verb–preposition collocations in Chinese makes it challenging for Chinese EFL learners to judge the correctness of “arrive under”. The structural inconsistency of certain word pairs complicates the processing of non-collocations for Chinese EFL learners, amplifying the differences in processing collocations and non-collocations in English.

### 5.2. The Role of Input Frequency and L2 Proficiency

For the main effect of input frequency, the results revealed that Chinese EFL learners exhibited sensitivity to the input frequency of collocations during language processing, as evidenced by the higher accuracy rates in judgments for high-frequency collocations compared to low-frequency ones. The finding contradicts Wray’s (2002) claim that L2 learners have limited sensitivity to collocation frequency. Instead, it supports Bybee’s (2007) “frequency effect”, which includes three primary effects of frequency: the maintenance effect, the reduction effect, and the autonomy effect. This study confirms the maintenance effect, where the repeated use of linguistic units reinforces their representations in memory, as well as the autonomy effect, in which high-frequency units are accessible under context-independent conditions. Increased frequency solidifies the entrenchment of corresponding linguistic structures, enabling the component words of collocations to be represented as cohesive units within the cognitive system, thereby enhancing the degree of holistic representation. Consequently, high-frequency co-occurrences are more likely to be psychologically salient than low-frequency co-occurrences (Cangır et al., 2017; Durrant & Doherty, 2010; Fioravanti et al., 2024).

For the main effect of L2 proficiency, the results showed a distinction between the high- and low-proficiency Chinese EFL learners in collocational processing, with the high-proficiency Chinese EFL learners exhibiting shorter reaction times and higher accuracy rates. This within-group difference may be closely related to the level of L2 exposure. Low-proficiency Chinese EFL learners tend to have less L2 exposure than high-proficiency learners. For these learners, the memory trace of collocation may disappear before it is encountered again, which could impede the acquisition of implicit word-pair knowledge related to collocations (Durrant & Schmitt, 2010).

Input frequency and L2 proficiency interactively influence the representation of collocations. Processing differences among learners of varying proficiency levels are more pronounced for high-frequency collocations. For low-proficiency Chinese EFL learners, increased input frequency enhances the holistic representation of collocations, whereas high-proficiency Chinese EFL learners exhibit similar processing patterns for both high- and low-frequency collocations. These findings may be attributed to familiarity. Adolescent Chinese EFL learners primarily acquire collocations from classroom-based textbooks, leading to limited exposure to diverse forms of language input. In contrast, adult Chinese EFL learners, particularly in university settings, are exposed to a broader range of language input, which reduces their dependence on textbooks for collocational learning. Consequently, increased language exposure enhances their familiarity with collocations as holistic units, thereby reducing the frequency effect (Lu, 2024).

Building on the preceding discussion, the present study proposes that the representation of collocations in the L2 mental lexicon exists as a continuum between analytic and holistic patterns, which advances the dual-route pattern suggested by Carrol and Conklin (2014). The increasing input frequency and improving L2 proficiency facilitate a transition toward a more holistic representation of collocations. This reflects a dynamic interplay between analytic retrieval and holistic recognition, with language experience serving as a key factor in shifting from the former to the latter. From a language development perspective, the holistic pattern of collocational representation gradually becomes entrenched in the mental lexicon of Chinese EFL learners.

## 6. Conclusions

This study employs a corpus-based psycholinguistic research paradigm to investigate how input frequency and L2 proficiency influence the representation of L2 collocations. The findings indicate that collocations possess a certain degree of psychological reality, with both input frequency and L2 proficiency significantly affecting the representation of collocations in the L2 mental lexicon of Chinese EFL learners. As input frequency and L2 proficiency increase, collocational representation tends to transit from analytic retrieval to holistic recognition, exhibiting a continuum pattern. It is worth noting that this study is limited by its exclusive focus on adjective–noun collocations, the lack of explanatory quality analysis, and the reliance on CETCL-derived collocations for Chinese EFL learners. Therefore, future research could address these limitations to provide a more comprehensive understanding of the representation of L2 collocations.

## Figures and Tables

**Table 1:** Biographical data for participants in the phrasal decision task (standard deviation in parentheses).

Group	N	Age	Gender (M/F)	SAL	LOS (Year)	LOR (Month)	OQPT Score
LNNS	32	17.16 (0.44)	11/21	7.97 (1.47)	10.19 (1.42)	0.03 (0.17)	34.05 (3.25)
HNNS	34	21.15 (0.66)	2/32	8.35 (1.20)	12.79 (1.20)	0.00 (0.00)	43.37 (3.34)

Note. N = number of participants; SAL = starting age of learning English; LOS = length of studying English through formal education; LOR = length of residence in English-speaking countries (periods shorter than one month being counted as none); OQPT = Oxford Quick Placement Test.

**Table 2:** Description of research items in the phrasal decision task.

Type	Frequency	Number	Example
Collocations	High	30	correct form
	Low	30	similar concept
Non-collocations	——	60	deeply hole

Note. The symbol of “— —”denotes the absence of frequency.

**Table 3:** Mean reaction times (in milliseconds) and accuracy rates (in percentage) with their standard deviations in parentheses.

Group	Type	Frequency	RTs (ms)	ACCs (%)
LNNS	Collocation	High	1162.03 (468.84)	94.27 (23.25)
	Collocation	Low	1391.58 (493.75)	89.48 (30.70)
	Non-collocation	——	1681.07 (598.86)	78.75 (40.92)
HNNS	Collocation	High	1042.09 (370.08)	97.94 (14.21)
	Collocation	Low	1158.09 (380.61)	95.00 (21.81)
	Non-collocation	——	1553.27 (518.96)	82.55 (37.96)

Note. The symbol of “— —”denotes the absence of frequency.

**Table 4:** Results of the linear mixed-effects model Ⅰ (collocation as reference categories).

**Random Effects**		**Variance**	**SD**		
Participant (Intercept)		0.05	0.23		
Item (Intercept)		0.04	0.19		
Residual		0.05	0.23		
**Fixed Effects**	**Estimate**	**Std. Error**	**df**	**t Value**	**Pr (>|t|)**
(Intercept)	6.96	0.03	159.20	220.41	<0.001
Type	0.47	0.01	5660.35	39.83	<0.001

**Table 5:** Results of the generalized linear model Ⅰ (collocation as reference categories).

	Estimate	Std. Error	z Value	Pr (>|t|)
(Intercept)	2.80	0.07	40.98	<0.001
Type	−1.36	0.08	−17.22	<0.001

**Table 6:** Results of the linear mixed-effects model Ⅱ (HNNS and high-frequency collocations as reference categories).

**Random Effects**		**Variance**	**SD**		
Participant (Intercept)		0.04	0.19		
Item (Intercept)		0.10	0.31		
Residual		0.01	0.12		
**Fixed Effects**	**Estimate**	**Std. Error**	**df**	**t value**	**Pr (>|t|)**
(Intercept)	6.98	0.05	144.72	144.43	<0.001
Group	0.13	0.05	64.84	2.66	0.01
Collocation frequency	−0.01	0.01	3548.00	−1.17	0.24
Group × Collocation frequency	0.08	0.01	3481.00	10.68	<0.001

**Table 7:** Results of the generalized linear model II (HNNS and high-frequency collocations as reference categories).

	Estimate	Std. Error	z Value	Pr (>|t|)
(Intercept)	3.93	0.24	16.10	<0.001
Group	−1.29	0.28	−4.59	<0.001
Collocation frequency	−1.44	0.31	−4.62	<0.001
Group×Collocation frequency	1.13	0.37	3.08	0.002

## Data Availability

The datasets generated during and/or analyzed during the current study are available from the corresponding author upon reasonable request.

## Supplementary Materials

The following supporting information can be downloaded at: https://www.mdpi.com/article/10.3390/bs15010046/s1, Table S1: Personal Information Questionnaire & Informed Consent Form. Table S2: List of Items Used in Phrasal-decision Task.
