# Supplementary material for: The Influence of Input Frequency and L2 Proficiency on the Representation of Collocations for Chinese EFL Learners
Source: Behav Sci (Basel). 2025 Jan 4;15(1):46. doi: 10.3390/bs15010046 (PMC11763040; doi:10.3390/bs15010046)
Supplement: Supplementary file 1 [file behavsci-15-00046-s001.zip › behavsci-3330030-supplementary.pdf]

Table S1: Personal Information Questionnaire & Informed Consent Form

姓名[Name]：
性别[Gender]：
年龄[Age]：
年级[Grade]：
您几岁开始在课堂中学习英语？
[At what age did you begin learning English in the classroom]
您接受了几年英语课堂学习？
[How many years have you received English classroom instruction]
您在学习与生活中是否为右利手？
[Are you right-handed in both learning and daily life]
您是否有在英语国家学习或生活的经历？若有，请注明旅居时长（月份）。
[Have you had experience studying or living in an English-speaking country? If yes, please specify the duration (in months)]
您是否有除英语外的其他语种的课堂学习经历？若有，请注明您学习的语种。
[Do you have classroom experience in any language other than English? If yes, please specify the language(s) studied]

如果您愿意参与这项实验，请签下您的名字。
我的名字是　　　　　，我自愿参与这项实验。我的个人资料均属保密，有关我的实验数据仅研究者可见，且采用编号标识不透露具体姓名。在后续实验阶段，我有权随时终止参与实验。
[If you agree to participate in this experiment, please sign below.
My name is _______, and I voluntarily agree to participate in this experiment. My personal information will be kept confidential, and my data will be identified only by a number, not by my name. I have the right to withdraw from the experiment at any time during the subsequent stages.]

年　　月　　日
[Date]

Table S2: List of Items Used in Phrasal-decision Task

I. Item Used for Low-proficiency Adolescent Chinese EFL Learners

High-frequency Collocations	Low-frequency Collocations	Baseline Non-collocations	
useful word	French accent	deeply hole	never reaction
correct form	quick action	apply above	whole read
main idea	sharp knife	make believe	weak plan
last week	adult animal	realize voice	without ill
small group	wrong answer	laugh beside	obtain aware
next morning	local area	far society	fake follow
plastic bag	poor artist	store through	join problem
dusty window	many aspects	common study	turn rule
special day	lovely autumn	every treat	journey stay
second floor	crying baby	pretty order	soft ability
same meaning	missing letter	raise kinds	sports soon
short story	naughty bear	hard talent	slim lesson
green bean	empty bowl	part month	truth tell
warm clothes	medical care	walk pay	improve into
loud music	lazy children	attend against	wet speed
western art	first choice	protect off	success gain
cold outside	serious illness	cheap sleep	healthy down
English speaker	major effect	bitter change	further brief
fresh water	strange feeling	blind fruit	talk forward
red light	bright future	another grow	guess dish
high level	left side	solve habit	strong dirty
good reason	sweet potato	lucky tea	live advice
little girl	fast food	marry between	careful give
long time	great place	very student	less build
best friend	human history	before step	accept reduce
young people	extra money	control notice	large sunny
right thing	mental health	thick movie	broad price
global warming	middle class	various would	arrive under
new book	recent year	campus will	range upon
bad news	other country	college rain	trust react

II. Item Used for High-proficiency Adult Chinese EFL Learners

**High-frequency Collocations**	**Low-frequency Collocations**	**Baseline Non-collocations**	
correct form	urban area	deeply hole	never reaction
native speaker	final product	apply above	whole read
wild animal	urgent need	make believe	weak plan
plastic bag	safe distance	realize voice	without ill
real world	bright future	laugh beside	obtain aware
next month	famous film	far society	fake follow
daily life	sunny day	store through	join problem
small group	dark room	common pay	turn rule
literary work	clear picture	every treat	journey stay
basic color	major finding	pretty order	soft ability
useful word	poor family	raise kinds	sport soon
polar bear	strong desire	hard talent	slim lesson
second floor	past decade	part market	truth tell
same meaning	similar concept	walk pay	improve into
last sentence	tall building	attend against	wet speed
direct action	moral value	protect off	success gain
main idea	current study	cheap sleep	healthy down
first step	large piece	bitter change	further brief
various ways	special kind	blind fruit	talk forward
short story	unknown author	another grow	guess dish
front door	left side	solve habit	window dirty
little girl	total number	lucky tea	live advice
long time	big mistake	marry between	careful give
best friend	direct result	very student	less build
young people	sweet potato	before gift	accept reduce
public school	great place	control notice	wide sunny
recent year	happy ending	thick movie	broad price
global warming	white shirt	quickly weather	arrive under
mobile phone	extra money	campus will	range upon
fast food	key factor	college rain	trust react
